# Estrogen Effects on the Mammary Gland in Early and Late Life and Breast Cancer Risk

**DOI:** 10.3389/fonc.2017.00110

**Published:** 2017-05-26

**Authors:** Genevieve Victoria Dall, Kara Louise Britt

**Affiliations:** ^1^Peter MacCallum Cancer Centre, Melbourne, VIC, Australia; ^2^The Sir Peter MacCallum Department of Oncology, University of Melbourne, Melbourne, VIC, Australia

**Keywords:** parity, breast cancer risk, menarche, menopause, estrogens

## Abstract

A woman has an increased risk of breast cancer if her lifelong estrogen exposure is increased due to an early menarche, a late menopause, and/or an absence of childbearing. For decades, it was presumed that the number of years of exposure drove the increased risk, however, recent epidemiological data have shown that early life exposure (young menarche) has a more significant effect on cancer risk than late menopause. Thus, rather than the overall exposure it seems that the timing of hormone exposure plays a major role in defining breast cancer risk. In support of this, it is also known that aberrant hormonal exposure prior to puberty can also increase breast cancer risk, yet the elevated estrogen levels during pregnancy decrease breast cancer risk. This suggests that the effects of estrogen on the mammary gland/breast are age-dependent. In this review article, we will discuss the existing epidemiological data linking hormone exposure and estrogen receptor-positive breast cancer risk including menarche, menopause, parity, and aberrant environmental hormone exposure. We will discuss the predominantly rodent generated experimental data that confirm the association with hormone exposure and breast cancer risk, confirming its use as a model system. We will review the work that has been done attempting to define the direct effects of estrogen on the breast, which are beginning to reveal the mechanism of increased cancer risk. We will then conclude with our views on the most pertinent questions to be addressed experimentally in order to explore the relationship between age, estrogen exposure, and breast cancer risk.

Breast cancer remains one of the most prevalent diseases in the western world, with one in eight women predicted to be affected by breast cancer in their lifetime. Improvements in detection, anti-estrogen therapies, and cytotoxic chemotherapy have led to increased survival rates, from 72% in 1980s to 89% in 2010. Despite this, the incidence of breast cancer has increased over the same period (1, 2). This year in Australia, 15,930 women are predicted to be diagnosed with breast cancer and this figure is expected to rise to 17,210 women by 2020. Similar increases in incidence have been reported in America, the United Kingdom, and China (3–5). Estrogen receptor (ER)-positive breast cancers are the only subtype that are increasing (6–9) and as they make up 75–80% of all breast cancer cases this may explain the increase in breast cancer incidence overall. It has been postulated that the rising incidence of ER+ breast cancer is driven by hormonal risk factors such as low and/or late parity, early menarche, late menopause, as well as the use of combined oral contraceptive (OCP) pill and postmenopausal hormone replacement therapy (HRT) (10–13) rather than determinants such as BMI and BRCA1/2 status. In this review we will discuss the epidemiological data and experimental models used to investigate the effects of these different hormonal factors on ER+ breast cancer risk.

## Parity

Childbearing and a lack thereof have been known to influence breast cancer risk ever since Bernardino Ramazzini documented an increase in breast cancer incidence in nuns in the eighteenth century (14). Over the last century, these observations have been supported by numerous epidemiological studies (15–17). Depending on the age at which childbearing begins, after a transient increase risk period immediately following pregnancy (18), parity provides lifelong protection against breast cancer by up to 50% (19). In the last two decades, large case–control and meta-analysis studies have shown that the protection provided by parity is restricted to hormone receptor positive tumors (ER+PR+) (20–22). Recent studies have attempted to investigate whether parity reduces the risk of different molecular subtypes of breast cancer but have shown conflicting results (23–25) highlighting the need for larger studies to shed light on these inconsistencies.

The older a mother is at the age of the first full-term birth, the less the protection that is instilled by the pregnancy (18, 20, 26–28). After 35 years of age, parity paradoxically increases risk compared to women who have not had children. An increasing number of births also confer protection (29–33) with additional births providing an extra 10% reduction in risk (34). In addition, the spacing between births can influence breast cancer risk, with less than 1 year or greater than 3 years providing more protection than a birth space of 1–2 years (35). The protective effect of childbearing is important to consider in relation to current reproductive trends. Recent reports have identified that more women in western cultures are remaining childless or delaying childbearing until after 35 years of age (1, 2, 36). In 2012, up to 20% of Australian women were childless, and of those that were bearing children, 24% had their first child after 35 years of age (37). It is, therefore, proposed that this decline in childbearing and increasing age at first full-term birth may be contributing to the rise in breast cancer incidence.

Considering the influence of age at first birth, number of births, and birth spacing on breast cancer risk, it is not surprising that breastfeeding is also able to modulate risk (38, 39). The reduction in breast cancer risk offered by breastfeeding is 4.3–4.5% for every 12 months of breast feeding (40, 41), a reduction that is in addition to the reduced risk following each birth. Despite these findings and a recommendation from the National Health and Medical Research Council to breastfeed for at least 6 months (42), only 50% of Australian babies were being breastfed at 4 months and this dropped to 29.7% at 9–12 months of age (43). In contrast to the protective effects of childbearing, the protection conferred by breastfeeding is not limited to ER+ breast cancer (20, 44, 45). The mechanisms of breastfeeding-induced protection are largely unknown, as are the reasons why its effects are not restricted to ER+ cancers.

In addition to the epidemiological studies in women, parity-induced protection against breast cancer has also been shown experimentally through the use of rodent models. Rodents have been used due to the similarities in morphological structure between the mammary gland and human breast and the conservation of genes and pathways between rodent and human mammary epithelial cell subpopulations (46). Parity in rodents reduces the incidence of carcinogen-induced mammary tumors (47, 48) and, as with women, shows a dependence on age, with younger mothers showing a greater reduction in tumor incidence (49, 50). The rodent models have also shown that the protective effects of pregnancy can be simulated through the administration of pregnancy levels of estrogen and progesterone to rodents (51, 52). This provides supporting evidence that parity-induced protection may be hormonally driven, and thus may explain why its protection is restricted to ER+ breast cancers. The mechanisms underlying parity-induced protection remain an active area of research with investigators assessing the role of the mammary stem cells, ER+ cells and other growth factors (53, 54).

## Menopause

Menopause is defined as the final menstrual period. It occurs when there has been a change in a woman’s reproductive hormones and the ovaries no longer release any eggs. Menopause itself is not a breast cancer risk factor, but over 70% of all breast cancer diagnoses are made in women who are 50 or older, and thus postmenopausal women have a higher risk than premenopausal women. The timing of menopause has been shown to significantly affect breast cancer risk. While not documented historically, the duration of reproductive years was identified as a breast cancer risk factor in early epidemiological studies (15, 55). Large-scale case–control studies and meta-analyses have now consistently shown that younger age at menopause decreases ER+ breast cancer risk (10, 56–59), with each year older at menopause increasing the risk by 2.9–4% (10, 59).

The age at which a woman undergoes menopause varies considerably between and within ethnicities (60); however, mother–daughter and twin studies have found that only 44–63% can be accounted by heritability (61–63). Mother and daughter studies have postulated that the heritability may be driven by genetic changes in hormone expression as the maternal age at menopause was found to be a strong predictor for high follicle-stimulating hormone (FSH) levels (an indicator of ovarian aging) in daughters (64). Genetic studies have tried to ascertain what may be mediating the timing and found that polymorphisms within the ER gene and ER signaling pathway are significantly associated with age at natural menopause (65, 66). It is not clear whether these polymorphisms are associated with increased or decreased ER signaling. Larger-scale genome-wide sequencing studies (between ~3,000 and 40,000 women included compared to ~200–900 in previous studies) have confirmed this association and have also reported further polymorphisms in DNA damage and repair genes and genes associated with mitochondrial DNA, FSH, and immune components (67–70). Together, these studies only explain ~4% of the variation in age at menopause. Furthermore, only one single nucleotide polymorphism (rs2517388) for age at menopause was associated with breast cancer risk (71). The rs2517388 polymorphism is located within a gene that encodes for a subunit of the MLL histone methyltransferase protein (72). MLL has been shown to act as a coregulator of ER-induced progesterone receptor gene activation (73). It is also involved in estrogen-dependent activation of kinesins (74), which have been linked to tamoxifen resistance. This may indirectly explain the link between the polymorphism, age at menopause, and breast cancer; however, this has not been tested functionally. Thus, the underlying reason why age at menopause affects breast cancer risk is still unknown. Women with a later menopause have been shown to have longer mean menstrual cycle length (75) than those with an average age menopause. It is not known what mediates this, but it is intriguing to think that it may be related to hormone levels in the follicular phase of the menstrual cycle seeing as the follicular phase length drives total cycle length.

Mouse models exploring the effects of menopausal age on breast cancer risk are challenging as mice do not undergo natural menopause. This may explain the lack in experimental data exploring or even confirming the abovementioned polymorphisms associated with age at menopause in animal models. However, like humans, menopause can be induced in mice by surgical removal of the ovaries. Using such a system one group have assessed the effect of timing of HRT on the postmenopausal mammary gland in an effort to explain the increase in breast cancer risk observed following HRT (76). They found that the postmenopausal gland (5 weeks post ovariectomy) was more responsive to estrogen-driven proliferation compared to their model of peri-menopause (immediately following ovariectomy). Similar studies could be performed to shed light on the underlying mechanism of an early versus late menopause as being protective against breast cancer incidence.

## Menarche

Age at menarche, like menopause, is also associated with breast cancer risk. However, unlike age at menopause, the older a woman is at age at menarche, the lower her risk of breast cancer. Several groups have now shown that starting menses prior to 11 years of age increases the risk of breast cancer, while a later age at menarche (14 years) reduces the risk (10, 58, 59, 77–81). Sisti and colleagues showed that the relative risk of breast cancer was increased by 5% for each year younger at menarche (78) and the Collaborative group on hormonal factors reported up to an 18% reduction in risk in those girls experiencing a late menarche (≥ 13), compared to those who began cycling at 11 (10).

During the last decade, epidemiological studies reporting on trends in age at menarche have shown that irrespective of ethnicity, the average age of menarche is ~12 years (10, 82–84). Historically, age at menarche was much older (85). A review published in 1982 assessing reports on age at menarche including 220,037 European women from 1795 to 1981 observed a 2–3 month decline in age at menarche per decade (86) with some reports finding the age at menarche to be as late as 16.5 years in 1840. They also observed a similar decline (2 months per decade) in US reports from 1877 to 1947. A more recent study assessing a cohort of 94,170 British women found a decline in age at menarche from 13.5 for girls born between 1908 and 1919 to 12.3 for girls born between 1990 and 1993 (84). This decline in age at menarche has also been observed in macaque colonies (87, 88) indicating that rather than being an effect of evolution, the decrease is due to environmental influences. Both the earlier and more recent human studies noticed that the rate of decline in age at menarche slowed in 1940s and has now been fairly consistent (average of 12 years of age) over the past 70 years. It is believed that this plateau in menarcheal age is due to improved nutrition and life quality. This is an important finding as the breast cancer incidence has continued to increase rapidly over the past century suggesting that early age at menarche is not the major reproductive factor influencing breast cancer incidence.

Factors that have been shown to affect the age at menarche include gestational exposure to smoke (89), diet (90), psychological state (91, 92), and BMI (93–95). The effect of BMI on age at menarche has not only been supported by numerous epidemiological findings but has also been shown experimentally in rhesus monkeys (87) and confirmed by genome-wide sequencing (96, 97). In one sequencing study, 30 new loci associated with age at menarche were identified, most of which have no clear function individually, but pathway analysis classified them into two groups, lipid metabolism and gene expression/cellular growth (96). While poor dietary choices contributing to BMI is considered an environmental factor influencing age at menarche, the effect of BMI on age at menarche has also been shown to be due to heritable factors (98). Indeed, excessive maternal weight gain during gestation has been shown to lower the age at menarche in daughters (99, 100). In concert with this, small-scale studies have shown that increased gestational weight gain leads to greater chance of obesity in adolescent offspring (101, 102), which then is known to influence the age at menarche (89, 93–97). Cumulatively, these data indicate that alterations during critical developmental points can actually determine a daughter’s weight which then influences her age at menarche and then in turn her breast cancer risk later in life.

It is unexpected that genome-wide sequencing studies did not find a strong association of estrogen-regulated genes and pathways with age at menarche, as was shown in the age at menopause studies (67, 96, 97). Certainly, menarche begins in response to ovarian hormones including estrogen, and higher levels of urinary estrogens have been observed in girls experiencing precocious menarche (103). This may be a key link to the influence age at menarche has on breast cancer risk, as if higher estrogen levels correlate with earlier age at menarche, it may be that the increase in breast cancer risk associated with younger ages at menarche is estrogen driven.

Despite the identification of candidate genes involved in the timing of menarche, experimental work to define the mechanism/s underlying pubertal timing has been limited. Most studies have been restricted to exploring the effects of environmental determinants such as exposure to seasonal changes (although the effect on age at menarche is thought to be a by-product of changes in growth), diet, and social status. This is due to the required use of macaques and non-human primates who also undergo a defined menarche (104). Of the 19 single nucleotide polymorphisms that have been associated with age at menarche, only two have also been associated with breast cancer risk (71), and thus a lot is still unknown about both why a women undergoes menarche when she does and why this affects her breast cancer risk.

## Age at Menarche is More Influential than Age at Menopause on Breast Cancer Risk

The observation that lengthening the reproductive life of a woman, either by an earlier menarche or later menopause, increases the risk of breast cancer would suggest that the overall duration of the exposure to estrogen is underlying the risk. However, a recent meta-analysis of reproductive events and breast cancer risk has found that age at menarche may be more of a deciding factor on the risk than age at menopause (10).

It is common practice for all epidemiological studies assessing reproductive factors on breast cancer risk to consider both menarche and menopause. This is due in large part to the earliest epidemiological studies identifying an association between reproductive timing and breast cancer risk (15, 55). Reanalysis of these landmark studies using modern statistics confirmed the early associations and also assessed whether age at menarche or age at menopause had a greater influence on breast cancer risk. This report showed inconsistent effects of age at menarche but very consistent effect of age at menopause where younger age at menopause reduces risk of breast cancer across two cohorts of women (27). A similar finding was also reported a decade earlier finding age at menopause a greater influence on breast cancer risk among parous women (105). However, since these reports, the Collaborative group on Hormonal Factors in Breast Cancer published a meta-analysis of 117 previously reported epidemiological studies showing that while later age at menopause does increase the risk of breast cancer, each year earlier at menarche increases breast cancer risk more than each year later at menopause (10). The meta-analysis included 425,055 women (118,964 cases versus 306,091 controls) providing it with sufficiently more power than the earlier work with a maximum of 1,000 cases and controls. The Collaborative group on Hormonal Factors in Breast Cancer also noted that there is no relationship between age and menarche and age at menopause, in that an earlier age at menarche does not influence the age at menopause and *vice versa*. This has been identified in a number of epidemiological reports with Forman and colleagues meta-analysis observing that of 36 studies investigating age at menarche and menopause, just 12 found a significant association between the 2 (60). This lack of an association has also more recently been supported by genome-wide sequencing analysis (70).

These findings contradict earlier theories that the influence of age at menarche and menopause on breast cancer risk was simply due to the duration of exposure to cycling ovarian hormones. Instead, it seems that the timing of the first exposure of the mammary gland to cyclic hormones sets up a developmental program that has consequences for breast cancer risk later in life.

## Aberrant Hormone Exposure is More Influential in the Young, Rather than Old Mammary Gland

Aberrant hormone exposures *via* clinical administration or natural exposure are known to increase the risk of breast cancer. Elevated endogenous hormones increase breast cancer risk; dizygotic twins can be exposed to up to two times the maternal estrogen levels that single pregnancies experience (106). In line with this studies have reported increased breast cancer risk in women later in life who belong to a dizygotic twin pair (106, 107).

Exogenous hormone exposure also modulates breast cancer risk with *in utero* exposure to synthetic estrogens, combined OCP use in young women, and HRT in postmenopausal women all increasing breast cancer risk. Maternal use of synthetic estrogen diethylstilbestrol, which was widely prescribed in 1940–1960s to prevent pregnancy complications, has been shown to significantly increase the risk of breast cancer in offspring (108, 109), but not until after the age of 40. Combined OCP use (estrogen + progestin) increases the risk of breast cancer (12, 110, 111) and like early age at menarche, the younger a woman is at the start of use, the higher her risk of breast cancer. One large meta-analysis reporting on 54 studies including 53,279 cases and 100,239 controls observed an RR of 1.6 (SD 0.142, *p* = 0.0001) for women who began OCP use before the age of 17 compared to an RR 1.2 (SD 0.047) in women commencing treatment after 22 years of age. The longer the duration of OCP also further increases the risk of breast cancer (112, 113). However, unlike age at menarche and *in utero* aberrant hormonal exposures, the increased risk of breast cancer from OCP is not lifelong and disappears between 4 and 10 years after use ceases (12, 110–113). Similarly, HRT use (estrogen and progestogens) in postmenopausal women increases the risk of breast cancer [adjusted RR = 2.0 (95% CI 1.8–2.12)] (114) and the risk increases with longer duration of use (11, 115). Like OCP use, the increased risk observed during HRT returns to baseline levels within 1–5 years (11, 114) but unlike OCP use, earlier age at first use is not associated with a higher increase in incidence.

Very few studies have been performed to experimentally explore the stimulatory effect of exogenous hormonal exposure on the normal mammary gland. In regard to HRT, as mentioned, rodent studies are complicated by the fact that rodents do not undergo natural menopause. Despite this, two studies have been performed assessing the effects of either estrogen alone (76) or estrogen in combination with progesterone (116) and both found a stimulation in proliferation in the mammary gland. However, estrogen alone does not increase the risk of breast cancer (117) and thus these findings cannot be readily applied to the epidemiological studies in women. Additionally, while estrogen combined with synthetic progestins is known to increase the risk of breast cancer, some studies have shown that estrogen with micronized progesterone does not (118, 119). Using macaques one group investigated the benefits of using micronized progesterone rather than synthetic progestins in HRT (120). They found increased lobular and ductal proliferation in those who received estrogen plus progestin compared to those receiving estrogen plus micronized progesterone. Transcriptional profiling of mammary tissue isolated from the two treatment groups showed a significant upregulation in the epidermal growth factor receptor/HER2 pathway and increased expression of proto-oncogene c-MYC (120). They did not see any changes in genes involved in ER signaling, which was unexpected given HRT increases the incidence of ER+ breast cancer. As these studies were performed in ovariectomized monkeys, their finding—while a significant advancement in our understanding of how aberrant synthetic hormonal exposure increases breast cancer risk—can only be applied to epidemiological findings on HRT and breast cancer risk, not OCP. To date, there have been no experimental studies on the effects of estrogen and progestin on normal mammary gland activity in ovary-intact mice.

Cumulatively, the epidemiological studies assessing aberrant hormonal exposures again point to the young mammary gland as being the most susceptible to hormone fluctuations and breast cancer risk modulation.

## What is Unique about the Young Mammary Gland That Makes it so Susceptible to Cancer Induction and Protection?

The fact that two crucial reproductive events, menarche and young age at parity, have the greatest effect on lifetime breast cancer risk suggests that the young mammary gland represents a crucial window in tumorigenic susceptibility. Why this is the case is less clear. Based on the epidemiological evidence for this, a few hypotheses have been generated, but again few have been tested experimentally, and this work is largely restricted to rodent models.

Mammary stem cells were originally proposed to play a role in the increased susceptibility to carcinogens in the young mammary gland. Russo and colleagues reported morphologically distinct structures within the mammary gland at different stages of transformation sensitivity, as ascertained through exposure to chemical carcinogens (48–50). Terminal end buds (TEBS) are club-like structures that facilitate the invasion of the mammary tree through the mammary fad pad in response to the onset of estrogen signaling at puberty (121). Once the buds reach the edges of the fat pad, they regress. Russo and colleagues quantitated the number and size of the TEBS and related the numbers to timeframes of susceptibility to the chemical carcinogen 7,12-dimethylbenz(*a*)anthracene (50). They showed that tumor incidence was highest when the TEB number and density was highest (49, 122). As it had been previously suggested that TEBs house mammary stem cells (123), they concluded that the density and number meant more mammary stem cells and thus a larger pool of transformation-sensitive cells. These data are compelling, however, support for mammary stem cells being housed or even enriched in the TEBs is conflicting (124–127). Furthermore, many groups have shown that mammary stem cells are not highest in number in the young mammary gland, but rather accumulate with age (127–129).

It has to also be considered that the high proliferative index of the mammary gland at puberty puts the cells at risk of obtaining and perpetuating deleterious mutations. As mentioned above, the pubertal mammary glands of rodents have a high content of TEBs, and it has been shown by several groups that the cells within these TEBs are highly mitotic (124, 130–133). During the process of the cell cycle, many checkpoints are in place to ensure the integrity of the DNA to be replicated and divided before mitosis can be completed. Should an error in DNA replication arise, DNA repair pathways are activated with varying levels of efficiency (134). Homologous recombination of double-stranded DNA breaks is considered the most effective repair mechanism, while non-homologous end-joining, although faster, is more prone to errors. Whether a normal cell chooses homologous recombination or non-homologous end joining to repair double-stranded DNA breaks is cell-cycle stage-specific (135–137). Homologous recombination is preferred for repair during S and G2/m phases as the machinery and DNA repair template required for homologous recombination are readily at hand. However, in the case of rapid proliferation, such as that seen at the onset of puberty in response to estrogen signaling (138), DNA replication stress occurs which may result in excessive amounts of homologous recombination. Ironically, this can lead to more mutations if misalignments (which are an infrequent but potentially detrimental consequence of homologous recombination) occur (139, 140). DNA replication stress can also lead to the selection of error-prone DNA damage repair mechanisms (141), and sometimes no repair at all. This results in potentially oncogenic mutations being passed onto daughter cells that then in turn may perpetuate the accumulation of more deleterious mutations. So, another hypothesis is that the earlier the surge of estrogen signaling at puberty, the earlier the start of rapid proliferation of mammary cells to generate the ductal tree and the more time the mammary gland has to accumulate these mutations that ultimately lead to tumor formation. Why this accumulation in mutations takes decades to reach a critical point at which they develop into tumors is unclear.

Switching cells from a proliferative program to a differentiation pathway is a common therapeutic avenue to prevent further growth of tumors (142–144). Pregnancy is the first time the mammary gland terminally differentiates. In the human this involves the conversion of immature type 1 mammary lobules (similar to the rodent TEBs) to the more differentiated type 3 lobules, while the acinar milk-producing units are considered a transient type 4 that arise briefly to facilitate lactation (145). Thus, parous mammary glands are comprised mostly of type 3 lobules, while nulliparous mammary gland are predominantly made up of type 1 lobules, although they do contain a small number of type 2 and even sometimes type 3 lobules. Compared to type 1 lobules, type 3 lobules are relatively growth quiescent and thus not contributing to potentially deleterious proliferative-induced DNA mutations. Therefore, should a woman undergo the terminal differentiation required for pregnancy at a young age, rather than remaining in a state of prolonged proliferation, her mammary tissue will be induced to become prematurely growth quiescent, and protected from oncogenic transformation.

## Conclusion and Future Perspectives

Overall epidemiological studies have revealed that age at menarche is a stronger determinant of breast cancer risk than age at menopause. Despite this, the trend for a decline in age at menarche has not been steadily changing while breast cancer incidence has continued to rise, indicating that age at menarche is not likely to be fueling the increase in breast cancer cases. Furthermore, one questions why if the age at menarche and age at menopause influence breast cancer risk, why are so few of the gene polymorphisms associated with these reproductive factors also related to breast cancer? Together the studies indicate that estrogen exposure is not the underlying link between menarche/menopause and breast cancer (because age at menarche has more of an influence). The work to date indicates that they influence breast cancer risk in different ways with menopause timing likely to be hormonally driven, whereas age at menarche might influence breast cancer risk indirectly through BMI. Increasing epidemiological evidence for these theories is emerging but functional studies are lacking.

By contrast, childbearing trends are mirroring breast cancer incidence. Increasing numbers of women today remain nulliparous, women are having fewer children and a quarter of new mothers are delaying the start of childbearing (2, 36, 37). These reproductive changes over the last century correlate with the increased breast cancer incidence over the same period. Functional studies have been performed using rodent models to show that the protection afforded by parity against breast cancer is hormonally driven and may involve mammary stem cells (53, 146), but are still yet to delineate the exact mechanisms of how undergoing a full-term pregnancy equips the mammary gland with protection against carcinogenesis.

The studies reviewed herein show that age at menarche and timing of pregnancy have the greatest influence on breast cancer risk indicating that this early window of life is the most sensitive. The protection afforded by parity does not affect all women and unfortunately we have no way to identify those who are protected by childbearing. It is intriguing to postulate that the women with the potential to receive the protective effects of parity may be the same women who are most at risk of a carcinogenic insult. It may be that their mammary glands are more sensitive to developmental programming, be it protective or detrimental. The important questions remaining are can we predict who these women are, and can we use this information to develop preventive therapeutics? Through further functional validation of the genetic pathways involved in age at menarche, age at menopause and parity, the field may step a little closer to understanding these complex reproductive systems and their effects on breast cancer risk.

## Author Contributions

The authors conceived and assisted in the writing of this review article.

## Conflict of Interest Statement

The authors declare that the research was conducted in the absence of any commercial or financial relationships that could be construed as a potential conflict of interest.
